# microRNA-124 Inhibits Migration and Invasion by Down-Regulating ROCK1 in Glioma

**DOI:** 10.1371/journal.pone.0069478

**Published:** 2013-07-23

**Authors:** Liwen An, Yongjun Liu, Anhua Wu, Yifu Guan

**Affiliations:** 1 Key Laboratory of Medical Cell Biology, Ministry of Education, Department of Biochemistry and Molecular Biology, China Medical University, Shenyang, China; 2 Department of Neurosurgery, Fourth Affiliated Hospital, China Medical University, Shenyang, China; 3 Department of Neurosurgery, First Affiliated Hospital, China Medical University, Shenyang, China; NIH/NCI, United States of America

## Abstract

**Background:**

The extraordinary invasive capability is a major cause of treatment failure and tumor recurrence in glioma, however, the molecular and cellular mechanisms governing glioma invasion remain poorly understood. Evidence in other cell systems has implicated the regulatory role of microRNA in cell motility and invasion, which promotes us to investigate the biological functions of miR-124 in glioma in this regard.

**Results:**

We have found that miR-124 is dramatically downregulated in clinical specimen of glioma and is negatively correlated with the tumor pathological grading in the current study. The cells transfected by miR-124 expression vector have demonstrated retarded cell mobility. Using a bioinformatics analysis approach, rho-associated coiled-coil containing protein kinase 1 (ROCK1), a well-known cell mobility-related gene, has been identified as the target of miR-124. A dual-luciferase reporter assay was used to confirm that miR-124 targeted directly the 3′UTR of ROCK1 gene and repressed the ROCK1 expression in U87MG human glioma cell line. Furthermore, experiments have shown that the decreased cell mobility was due to the actin cytoskeleton rearrangements and the reduced cell surface ruffle in U87MG glioma cells. These results are similar to the cellular responses of U87MG glioma cells to the treatment of Y-27632, an inhibitor of ROCK protein. Moreover, a constitutively active ROCK1 in miR-124 over-expressed glioma cells reversed the effects of miR-124. Our results revealed a novel mechanism that miR-124 inhibits glioma cells migration and invasion via ROCK1 downregulation.

**Conclusions:**

These results suggest that miR-124 may function as anti-migration and anti-invasion influence in glioma and provides a potential approach for developing miR-124-based therapeutic strategies for malignant glioma therapy.

## Introduction

Brain tumors account for ∼90% of all primary central nervous system tumors. In the United States, it is estimated 22,910 new cases of brain and related 13,700 deaths in 2012 [1]. Gliomas are the most common type of malignant primary brain tumor, accounting for 80% of malignant case [2], [3]. Due to its high invasive neoplasm infiltrating diffusely into regions of normal brain, glioma is extremely difficult to be cured by total surgical resection or radiotherapy, leading to a high recurrences and poor prognosis. Despite of multi-modality treatment, the median survival of patients suffering from malignant glioma such as glioblastoma multiforme (GBM) is only 12 to 15 months [4]. Hence, it is urgently needed to understand the mechanisms of glioma cell’s migration and invasion and develop more effective curative therapies.

MicroRNAs (miRNAs) are endogenous non-coding RNAs of approximately 21–23 nucleotides long. Being expressed in a tissue-specific manner during development of organisms, they regulate the gene expression by interacting specifically with 3′-untranslated regions (3′UTR) of mRNA, reducing the stability of mRNAs and leading to reduced expression of protein [5]. Since miRNA may have many targets, they play key roles to regulate many biological processes such as embryonic development [6], differentiation [7], proliferation [8], cell death [9] and autophagy [10]. Emerging evidence has strongly suggested that aberrant miRNA expression is a common feature of many human cancers, functioning as either tumor suppressors or oncogenes [11]–[16].

Previous researches have shown that miRNAs have a very close relationship with glioma development [17]–[24]. microRNA-124 (miR-124) is abundantly expressed in normal brain tissue [25], necessary for embryonic neuronal differentiation which has been widely investigated in physiological neural development [26] and is highly conserved across species. It regulates some proliferation-related genes such as cyclin-dependent kinase 6 [27], [28], aryl hydrocarbon receptor (AHR) [29], sphingosine kinase 1 (SPHK1) [30], androgen receptor(AR) [31], and solute carrier family 16, member 1 (SLC16A1) [32]. miR-124 has been documented as a tumor suppressor since low expression of miR-124 was observed in several types of human cancers [27]–[35]. However, biological impacts of miR-124 on glioma cell migration and invasion have seldom been published.

In the current study, we have observed that miR-124 was downregulated in malignant glioma and its expression was correlated negatively with the pathological grading of glioma. Furthermore, we have identified that miR-124 regulated the ROCK1 gene, and ROCK1 protein expression caused actin cytoskeleton rearrangements, reduced cell surface ruffle, and suppressed glioma cell invasion. A constitutively active ROCK1 in miR-124 over-expressed glioma cells reversed the effects of miR-124. Our findings uncovered an important role of miR-124 in glioma morphology, motility and invasion via ROCK1 for the first time. These experimental results facilitate our understanding about the mechanism of the invasive ability of human glioma cells and pinpointed therapeutic options for glioma treatment.

## Results

### miR-124 is Significantly Downregulated in Human Glioma Tissues

To determine the expression level of miR-124 in clinical tissue specimens, we collected sixteen primary glioma tissue samples and extracted their total RNA. These sixteen primary glioma samples were classified into four grades according to WHO standard [2]: three WHO grade I, five WHO grade II, five WHO grade III and three WHO grade IV. Quantitative real-time PCR (qRT-PCR) was performed to determine the expression of miR-124 with respect to the internal standard RNU6-2.

Considering the fact that the our clinical samples were obtained from elderly patients and the research reports that anaplastic astrocytoma (WHO grade III) shares the similar biomolecular expression pattern and poor outcome of glioblastomas in the elderly patients [36], [37], we divided clinical samples into two groups: low grade gliomas (grade I∼II, n = 8) and high grade gliomas (grade III–IV, n = 8).

**Figure 1:**
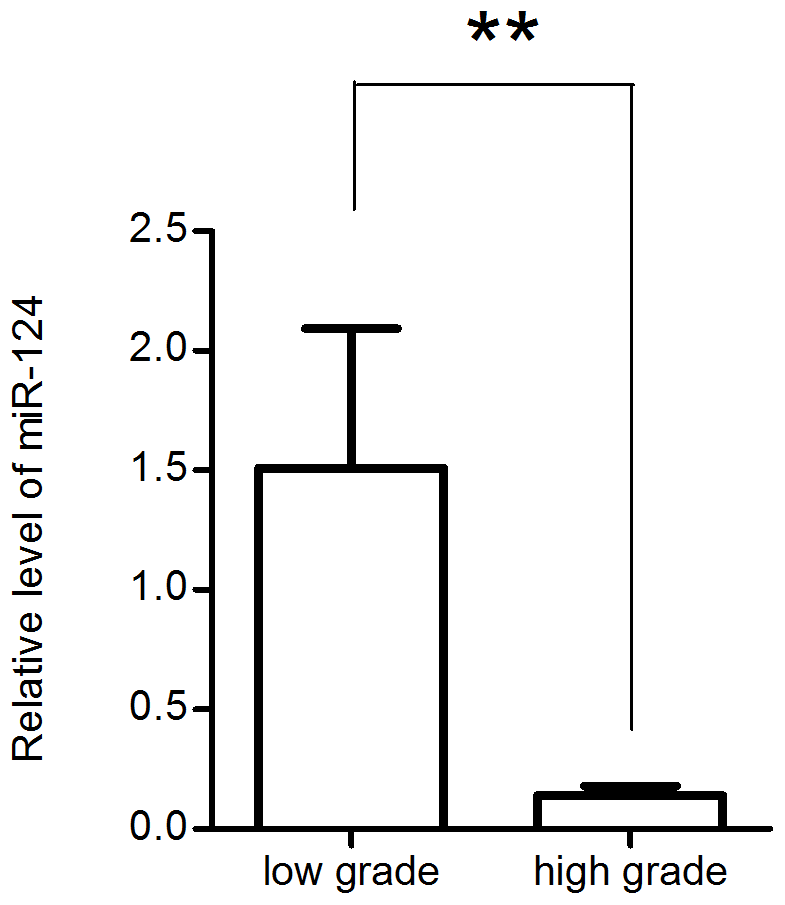
qPCR assays of miR-124 expression levels in glioma tissue samples. The expression level of miR-124 was downregulated significantly in high grade human glioma tissues (five grade III and three grade IV) than that in low grade human glioma tissues (three grade I and five grade II) determined using qRT-PCR.

The qRT-PCR results (Figure 1) showed that the expression level of miR-124 was significantly lower in high grade gliomas than that in low grade gliomas, demonstrating a negatively correlation of the endogenous miR-124 expression with the WHO grade (Spearman r = -0.5423, p<0.01). These data exhibits a good consistence with previous studies [38], [39]and indicates a putative tumor suppressor role for miR-124 in glioma.

### The Overexpressed miR-124 Inhibits the Cell Motility

**Figure 2:**
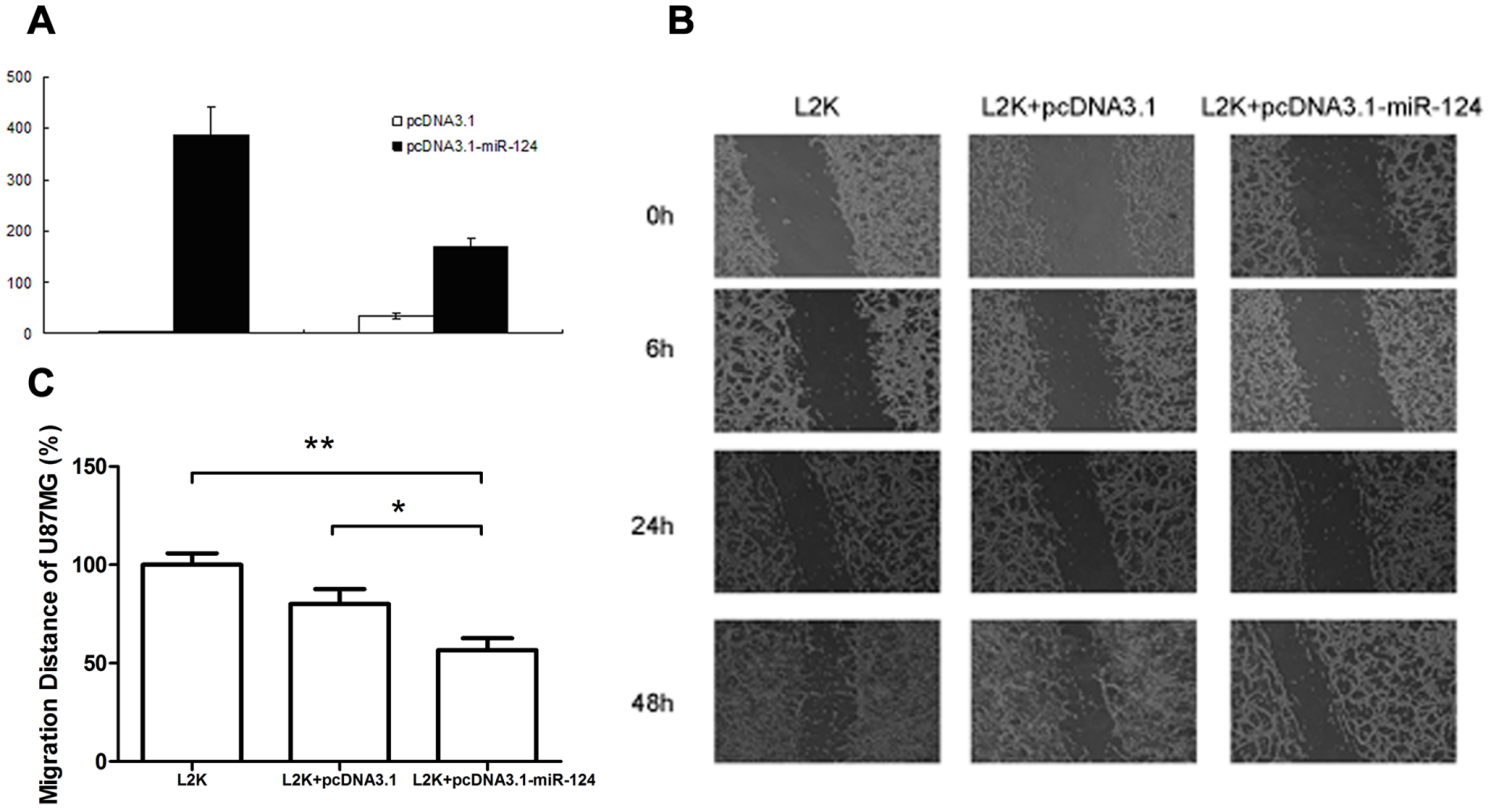
Evaluation of biological functions of miR-124 in transfected HEK293ET and U87MG cells. (A) The expression level of miR-124 was measured using qRT-PCR after miR-124 transfected HEK293ET and U87MG cells for 48 h. Data presented are mean values of three independent experimental results and compared with the level of miR-124 obtained in mock control (Lipofectamine2000 blank) that is normalized to 1. (B) Wound-healing assay of U87MG glioma cells transfected with either control or the miR-124 expression vector, respectively.

To decipher the biological function of miR-124 in glioma cells, we constructed a miR-124 expression vector (named as pcDNA3.1-miR-124) and transiently transfected into HEK293ET and human glioma cells U87MG and U251, respectively, to create a gain-of-function behavior in cell lines. Forty-eight hours later, the expression levels of miR-124 in all cell lines were examined using qRT-PCR. As shown in Figure 2A, the expression of miR-124 was enhanced significantly (by a factor of hundreds) in HEK293ET cells transfected with pcDNA3.1-miR-124 vector when compared with cells transfected with pcDNA3.1. The transfected U87MG cells (Figure 2A) and U251 cells (data not shown) demonstrated similar trend. All these results proved a success and effectiveness of miR-124 expression vector construction and transfection.

The migration ability of the transfected cells was further tested using the wound-healing assay. In this study, two controls were used: lipofectamine2000 blank (referred to as L2K) and lipofectamine2000 blank fused with pcDNA3.1 (referred to as L2K+pcDNA3.1). L2K+pcDNA3.1-miR-124 was used as the miR-124 expression vector.

The wound-healing process was monitored at 0, 6, 24 and 48 hours after scrape for U87MG cells. Images in Figure 2B and 2C clearly showed that the cells transfected with vector pcDNA3.1-miR-124 have a retarded mobility in comparison with other two controls, and similar results were also observed in U251 cells (Figure S1). These results indicated the anti-migration effect of miR-124 in glioma cells.

### miR-124 Interacts Specifically with the 3′UTR Region of ROCK1

**Figure 3:**
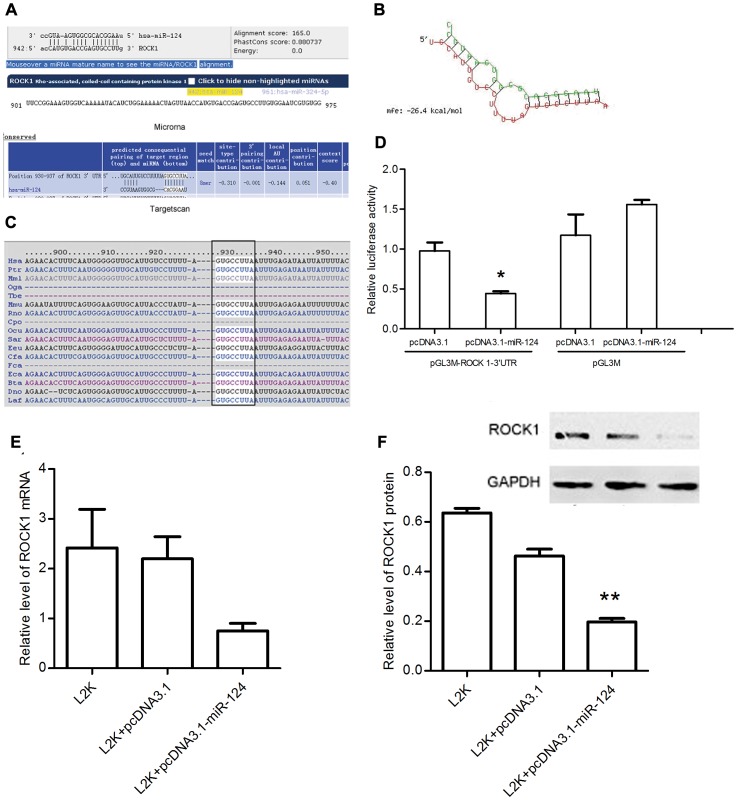
ROCK1 is target of miR-124. (A) Illustration of the predicted miR-124-binding sequences in the 3′UTR region of ROCK1. (B) The calculated free energy for hybridization of the ROCK1 3′UTR and miR-124 (Red color: ROCK1, Green color: miR-124). (C) Homology analysis of the 3′UTR sequences of 13 different species recognized by miR-124 seed sequence. (D) Luciferase analysis in HEK293ET cells. The assay was repeated three times with each assay being performed in three wells, and similar results were obtained each time. (E) qRT-PCR assay of ROCK1 levels treated with either pcDNA3.1 or pcDNA3.1-miR-124 for 48 h, compared with mock control in U87MG cells. (F) Western blot analysis of ROCK1 expression treated with either pcDNA3.1 or pcDNA3.1-miR-124 for 72 h, compared with mock control in U87MG cells.

After observing the altered cell mobility by miR-124, we started to search the potential genes involved in regulating the cell motility. Using TargetScan (Release 4.2) and miRanda (August 2010 Release) online searching programs, we have identified cell motility-related gene (ROCK1) as the potential target of miR-124. A 100% matched sequence was found at the nts 930–937 region of ROCK1 mRNA 3′UTR (NM_005406) (Figure 3A). The free energy (ΔG) was calculated about –26.4 kcal/mol for hybrid of ROCK1 3′UTR region and miR-124 by BibiServ analysis (Figure 3B). In addition, homology analyses have shown that the nucleotide sequences of 3′UTR of the ROCK1 gene targeted by miR-124 seed sequence were highly conserved among different species (Figure 3C).

To confirm the hypothesis that miR-124 targets the 3′UTR region of ROCK1, we cloned the vector that the entire 3′UTR region of ROCK1 was connected at the downstream of a modified luciferase reporter gene (named as pGL3M-ROCK1-3′UTR), and co-transfected the HEK293ET cells with this vector along with either the miR-124 expression vector or its negative control. In the case of pGL3M, the luciferase activities of cells transfected with either pcDNA3.1 or pcDNA3.1-miR-124 showed a slightly difference (by <16%). We found that co-transfection of miR-124 expression vector along with the full-length 3′UTR of ROCK1 caused a significant decrease by over 50% in luciferase units compared to controls (Figure 3D). These results demonstrated unambiguously that miR-124 targeted specifically the 3′UTR region of ROCK1.

Moreover, we transfected U87MG cells with either control or pcDNA3.1-miR-124, and determined the endogenous expression of ROCK1 at both protein and mRNA levels, respectively. qRT-PCR results revealed that although the mRNA level of ROCK1 was attenuated, it was not significantly affected by miR-124 in comparison with the control after statistics calculation (Figure 3E). However, the ROCK1 protein was remarkably reduced after transfection (Figure 3F). These data provided strong evidence that miR-124 suppressed ROCK1 gene expression by regulating 3′UTR at the post-transcription level.

### miR-124 Overexpression Suppresses the Glioma Cell Invasion by Affecting the Actin Cytoskeleton Rearrangements and Reducing Cell Surface Ruffles

Based on the inhibitory effects of miR-124 on ROCK1 protein expression and glioma cell locomotion, we reasoned that miR-124 may have an impact on cell invasive capacity.

**Figure 4:**
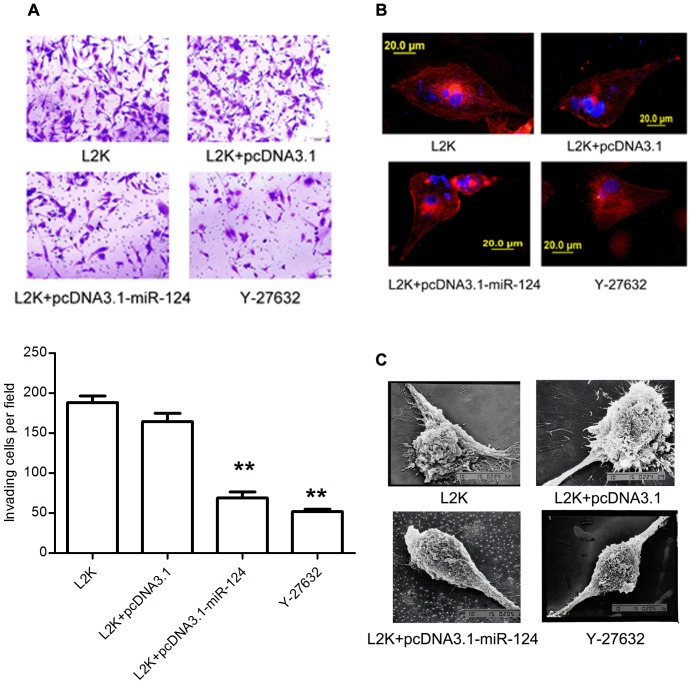
Identification of biological function of miR-124 in U87MG cells. (A) In vitro invasion assay. Invasive cells were stained and the average number of cells was counted at random six fields of vision. The data was an average value of three independent experiments. (Magnification: 100×; scale bars: 100 µm) (B) Stress fiber staining in U87MG glioma cells (Scale bars: 20 µm). (C) Scanning electron microscopy of U87MG cells treated with miR-124 expression vector or control and Y-27632 (Scale bars: 10 µm).

Matrigel invasion assays have been employed to assess the correlation of the miR-124 and the cell invasion. Forty-eight hours after transfection, a cell suspension was added into the upper chamber of matrigel-coated inserts. The lower chamber was filled with 500 µl of media containing 20% FBS and cultured for another 24 hours. Invasive cells were stained and the average number of cells was counted. Images of the stained cells demonstrated that ectopic overexpression of miR-124 significantly reduced the invasiveness of U87MG cells (lower left image, Figure 4A); the number of invasive cells able to digest the extracellular matrix and migrate through pores in the membrane was reduced by over 50%. Interestingly, this result was consistent with the result of Y-27632-treated cells (lower right image, Figure 4A), which has been known as a ROCK inhibitor.

It has been demonstrated that the Rho/ROCK pathway participates in regulating cytoskeletal signaling events and is crucial for cell motility. Moreover, cytoskeletal reorganization exemplified by the formation of stress fibre bundling arrays is essential for the contractile motion of cancer cells [40], [41]. We thus examined the status of stress fibre formation and polymerised actin in U87MG cells. Figure 4B showed the cytoskeleton changes inside cells. The actin filaments were stained with rhodamine phalloidin and examined by fluorescence microscopic. In the mock control or negative control U87MG cells (upper images, Figure 4B), the actin filaments were distribution presented as many dot-like and cluster-like actin assemblies spanning the length of the cells. However, the actin cytoskeletons of cells were completely different in the pcDNA3.1-miR-124 treated U87MG cells that they showed a decrease in the length and number of actin fibers in cells (lower left image, Figure 4B); do not have the same extent like the control groups. Similar phenotypic changes could be visualized in U87MG cells treated with 10 µM Y-27632, showed a loss of actin stress fibers, which has been reported with 50 µM Y-27632 [42].

Furthermore, filopodia and lamellipodia on cell membrane surfaces have been identified as dynamic cellular features, requiring actin polymerization/depolymerization for cancer cell invasion. We investigated the impact of miR-124-mediated cell morphology alternation in U87MG cells. Under scanning electron microscope, the negative control U87MG cells presented the elongated and fibroblast-like morphology with many filopodia and lamellipodia on the cell surfaces, probably for cell protrusion (upper images, Figure 4C). In contrast, higher miR-124 expression led to remarkable morphological changes in that cells became a round-like and shrunken form, and there was an obvious reduction of long and thin protrusions on the surface of cells transfected with pcDNA3.1-miR-124 (lower left image, Figure 4C).

These results furnished additional experimental evidence that miR-124 plays an important role in regulating cellular events related to cancer invasion. Furthermore, we used Y-27632 to treat U87MG cells, and observed that the diminished density and looser structure of the actin meshwork in U87MG glioma cells and the reduced protrusion by scan electron microscopy (lower right image, Figure 4C). All these results were similar to our in vitro observations in miR-124 overexpressioned cells, indicating a potential role of ROCK1 in glioma cell invasion.

### ROCK1 Partially Rescues the miR-124-induced Invasion Inhibition

**Figure 5:**
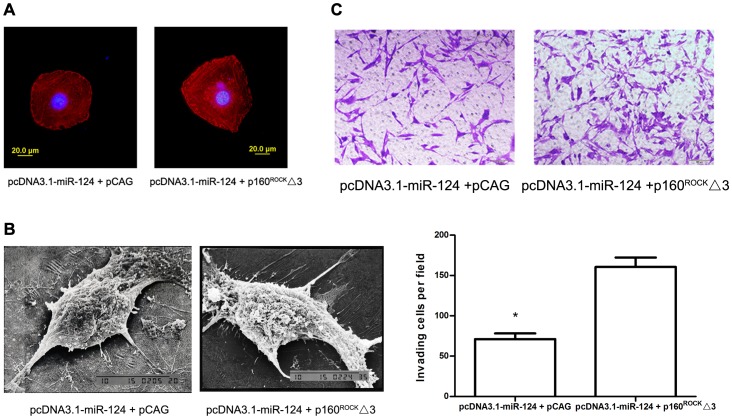
Reintroduction of ROCK1 rescues the miR-124-induced invasion inhibition. (A) Stress fiber staining in U87MG glioma cells treated with miR-124 expression vector along with p160^ROCK^Δ3 or control pCAG-myc (Scale bars: 20 µm). (B) Scanning electron microscopy of U87MG cells. (C) In vitro invasion assay. Invasive cells were stained and the average number of cells was counted at random six fields of vision. The data was an average value of three independent experiments. (Magnification: 100×; scale bars: 100 µm).

To further understand the role of ROCK1 in miR-124-mediated anti-invasion, we treated U87MG cells with pCAG-myc-p160^ROCK^Δ3 [43] (a constitutively active ROCK1, p160^ROCK^Δ3) or control pCAG-myc and co-transfected with pcDNA3.1-miR-124 followed by functional assays. Expression of these constructs was confirmed by western blot analysis for the myc-tagged antigen. As expected, ectopic expression of ROCK1 (without 3′UTR) significantly abrogated the miR-124-mediated anti-invasion by affecting the actin cytoskeleton rearrangements and reducing cell surface ruffles. The F-actin filaments stained with rhodamine phalloidin showed that ectopically expressing ROCK1 significantly increased the dot-like and cluster-like actin assemblies spanning the length of the cells (Figure 5A). Furthermore, the impact of miR-124-mediated cell morphology alternation in U87MG cells was restored compared to control pCAG-myc-treated cells, as observed after miR-124 suppression under scanning electron microscope (Figure 5B). Moreover, treatment with p160^ROCK^Δ3 also led to increased invasion of U87MG cells (Figure 5C). These results suggest that the anti-invasion effects of miR-124 are in part facilitated by ROCK1 downregulation.

## Discussion

Accumulating evidence has suggested that miRNAs are able to regulate multiple genes involving almost all aspects of cancer biology [13]. Investigation of these miRNAs would expand our view to better understand carcinogenesis by analyzing mRNA target associated and miRNA-mediated pathways. Several independent research groups have reported the correlation of alterations of miRNA expression with the gliomagenesis, patient diagnosing, potential prognosis and therapeutic tool [17], [44]–[45]. In this study, we focused on miR-124, a putative tumor suppressor in several human cancers. A variety of targets have been found to be regulated by miR-124, including proliferation-related genes [27]–[30], invasion/metastasis-related genes [33]–[35] and so on. It has been known that invasion, one of the most important hallmarks of malignant tumors [46], [47], is the incurable factor for human glioma. Study by Fowler et al [33] has reported that transfection of commercialized miR-124 precursor in GBM cell line A172 resulted in diminished cell migration and invasion as well as downregulated three targeted genes: Ras GTPase activating protein 1 (IQGAP1), cytoskeletal proteins laminin c1 (LAMC1) and integrin β1 (ITGB1). However, the median survival was not significantly different between the high and low miR-124 expression of GBM patients, which might be due to the extraordinary high malignancy of GBM. Xia et al [39] have reported the down-regulation of miR-124 in a larger patient clinical specimen, which included human glioma tissue samples (n = 27) and non-glioma patients samples (n = 20, two non-tumor brain tissues). They identified SNAI2, a member of the Snail family of zinc finger transcription factors - because it has been implicated in epithelial-mesenchymal transition (EMT) [48], [49] and tumor metastasis, as a direct functional target of miR-124. The enhanced miR-124 expression significantly inhibited glioma cell invasion using matrigel invasion assay and tumor xenografts in nude mice.

In this study, we identified that low expression of miR-124 was closely associated with a more aggressive tumor phenotype. Furthermore, the level of endogenous miR-124 is negatively correlated with the tumor pathological grading, indicating an association with the progression of glioma. Therefore, it is possible to be developed into a biomarker for diagnosis.

We performed functional analysis to examine the function of miR-124. Reintroduction of miR-124 through an expression vector dramatically repressed glioma cell migration and invasion in vitro. These findings suggest that miR-124 plays a critical role in the invasive potential of glioma. We also observed that the number of miR-124 transfected cells was reduced slightly in the wound-healing assay (Figure 2B). It could be due to either the serum depletion during the longer experimental duration or some unclassified functions of miR-124 in regulating other proliferation-related genes. In addition, by transfecting EGFP plasmid in the two glioma cell lines U87MG and U251, we identified that U87MG cells have a higher transfection efficiency than U251 (data not shown). Accordingly, we chose U87MG cells as the objective of the experiment.

As a next step, we used the on line websites to identify target genes of miR-124, interestingly, all of which are closely related to tumor migration and invasion. In general, cell movement is affected through a combination of protrusive and contractile events. Non-muscle cells contain stress fibres – bundles of approximately 10–30 actin filaments [50]. A large number of signaling proteins, scaffolding proteins and actin-binding proteins (ABPs) participate this very complicated biological process in a temporal-specific and spatial-specific manner, as well as in the cooperative fashion [51]. ROCKs have been shown to be a central player in the formation of stress fibers via phosphorylation of myosin light chain [40], [52]–[53]. Two ROCK isoforms have been identified: ROCK1 and ROCK2. Sequence analyses have shown a 65% sequence homology between these two ROCK isoforms, and in the kinase domains, their sequence similarity could be as high as 92% [54]. The ROCK2 transcript is highly expressed in muscle and brain tissues, whereas the ROCK1 is localized in non-nerves tissues [53]. Deregulation of Rho/ROCK signaling pathway has been reported across diverse tumors types [55], [56]. Several preclinical and clinical studies have utilized inhibitors of Rho/ROCK signaling pathway for anticancer therapeutics in prostate, lung, melanoma, and many other tumor types with remarkable success [57]–[59]. Using bioinformatics and experimental methods, we assessed ROCK1 as potential functional targets of miR-124. We performed a 3′UTR luciferase assay and observed that luciferase activity was decreased after co-transfection of the miR-124 expression vector and a 3′UTR vector containing the ROCK1/miR-124 target sequence. ROCK1 protein expression was also significantly downregulated in U87MG cells that were transfected with the miR-124 expression vector. All these data clearly indicate that ROCK1 is a direct target of miR-124.

It has been reported that as a cell moves in a designated direction, cooperation of continuous actin polymerisation and depolymerisation occurs simultaneously, which leads the cells to protrude at its anterior front. Concurrently, the cell undergoes consecutive actomyosin contractions and separates from the posterior end, and directional cell movement occurs. In addition, formation of the stress fibres, filopodia and lamellipodia also requires actin polymerisation. In this study, our results show that exogenous expression of miR-124 in U87MG cells substantially suppressed the formation of the stress fibres and cell protrusions. Previous studies have reported that downregulation of ROCK1 reduced markedly cell spanning F-actin fibers but enhanced the production of cortical fibers. On the other hand, downregulation of ROCK2 promoted the production of shorter intracellular fibers and destabilized the cortical F-actin fibers, leading to the formation of invaginations in human-derived renal proximal tubular cell line HKC-8 peripheral cells [60]. The changes in the F-actin localization in glioma cells treated with miR-124 in this study showed a good agreement (Figure 4B).

In addition to the changes of stress fibres, the invasion behavior of glioma was inhibited by up-regulation of miR-124. This phenomenon is consistent with the effect of ROCK inhibitor Y-27632 reported previously using GBM cell lines T98G and 8401 [61], [62]. These observations suggested that miR-124 retard the glioma cell migration and invasion by inhibiting the formation of the stress fibres via ROCK1 regulation. Notably, a constitutively active ROCK1 in miR-124 over-expressed glioma cells reversed the effects of miR-124, suggesting the biological role of preventive invasion of miR-124 due mainly to the ROCK1 down-regulation. However, contradictory evidence showed that the invasive and migratory properties of astrocytoma were enhanced by inhibition of ROCK [42], [63]. The inconsistence among these studies might be due to either the differences of cell lines, or the inhibitor dosages or the model systems used in these different studies. More systematic studies, thus, have been proposed to be performed by following the standard experimental approaches to identify the true functions of ROCK proteins in migratory and invasive phenotypes of glioma cells.

To extend these findings to glioma tissues, we also measured ROCK1 mRNA expression by qRT-PCR in the same clinical samples for miR-124 expression. Analyses confirmed that this difference was statistically significant between the low grade gliomas and the high grade gliomas (Figure S2). However, the correlation between the endogenous ROCK1 mRNA expression of clinical samples and the pathological grading was not established since we were unable to extract enough protein for the measurement. A study by Oellers et al [64] reported that ROCK was implicated in the migration of glioma cells on myelinated axons, a process that is still poorly understood. They also showed another important finding that ROCK1 was much less expressed while ROCK2 was highly expressed in the normal brain tissue. In contrast, ROCK1 was highly expressed and ROCK2 expression was almost undetectable in three WHO grade III and three WHO grade IV gliomas using immunohistochemistry and western blotting. These data indicated that at least the ROCK1 isoform is expressed in malignant human gliomas with a high potential for migration and invasion although this conclusion needs to be confirmed via a large number of clinical samples. Various compounds such as Y-27632 have been found to inhibit ROCK and inhibition of ROCK by fasudil leads to beneficial effects in patients with cardiovascular disease clinically [65]. The question is that the inhibitors are non-isoform-selective. Thus, designing an inhibitor that specifically blocks ROCK1 and using a siRNA approach would further help to unravel its role in primary gliomas.

## Conclusions

**Figure 6:**
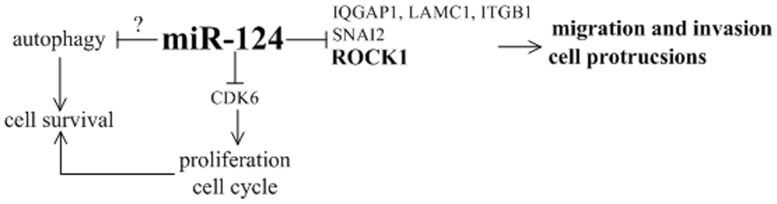
Proposed model of miR-124 function in glioma development and progression. The target of miR-124 in bold fonts was confirmed in this study.

In this study, we have demonstrated that the endogenous expression of miR-124 was negatively correlated with the tumor pathological grading of clinical glioma samples, and bioioformatic analyses have identified the target gene of miR-124. Further in vitro experiments have showed that miR-124 reduced migration and invasion, affected actin cytoskeleton rearrangements and reduced cell surface protrusion by suppressing the expression of ROCK1 protein. Moreover, a constitutively active ROCK1 in miR-124 over-expressed glioma cells rescued the effects of miR-124. Based on our observations and results reported by other groups, we have proposed a model to elucidate the potential roles of miR-124 in glioma (Figure 6). The experimental data and conclusions in the present study furnish valuable information regarding the biological functions of miR-124 and the possible mechanism of the migration and invasion of glioma tumor. Thus, further studies will be focused on the molecular network involved in the ROCK1 regulation on a large quantity of clinical sample basis.

## Materials and Methods

### Cells Lines and Reagents

HEK293ET and glioma cell lines U87MG and U251 (human) were purchased from the American Type Culture Collection (Manassas, VA, USA). All cell lines were cultured at 37°C with 5% CO_2_ in DMEM supplemented with 10% fetal bovine serum (FBS).

Original stock solutions of Y-27632 (Calbiochem, Nottingham, Germany) at a concentration of 5 mM was stored at -20°C and freshly dissolved in culture medium before use.

### Clinical Glioma Samples

Human glioma samples from surgery were collected from the neurosurgery department of the First Affiliated Hospital of China Medical University from adult patients, freshly resected during surgery. The samples were snap-frozen in liquid nitrogen and stored at −80°C for subsequent total RNA extraction. All human materials used were approved by the Ethical Committee in China Medical University. All participants have provided their written consent to participate in this study.

### RNA Isolation, Reverse Transcription and Quantitative Real-time PCR of mRNA and miRNA

Total RNA, including miRNAs, was isolated by TRIzol reagent (Invitrogen, Carlsbad, USA) following the manufacturer’s protocol. qRT-PCR were performed using ImProm-II™ reverse transcriptase (Promega, Madison, USA) and SYBR® Premix Ex Taq™ II (TaKaRa, Dalian, China), and detected with the ABI7500 Real-time PCR system instrument (Applied Biosystems, Foster City, CA, USA). For ROCK1 quantification with the internal control GAPDH, the primers for ROCK1 were AACCATGTGACTGAGTGCCC and TCAGTGTGTTGTGCCAAAGC. Primers for GAPDH were AATGGGCAGCCGTTAGGAAA and TGAAGGGGTCATTGATGGCA.

For quantification of miR-124, 10ng of RNA was used as a template and cDNA was synthesized with miRNA-specific primers, performed as described [66], [67], the miR-124 level was analyzed with internal control RNU6-2. Specific stem-loop reverse transcription primer and forward primer of PCR for miR-124 were CTCAACTGGTGTCGTGGAGTCGGCTACTAAGTTGGCGAGATTCA and ACACTCCAAGGGCTGTAACGGGTGCCGGAA. Primers for RNU6-2 were CTCAACTGGTGTCGTGGAGTCGGCAATTGACAAGTTGAAATATG and ACACTCCAAGGGCTGTAACGGGTGCCGGAA.

The expression level of ROCK1 and miR-124 was calculated by using 2^−ΔΔCt^ analysis method [68], normalized to the control group.

### Vector Construction

The human pre-miR-124 sequence was amplified and cloned into pcDNA3.1-hisA constructs (Invitrogen) to generate pcDNA3.1-miR-124 expression vector. The set of primers were GAGAATTCTTGCATCTCTAAGCCCCTGT and TCTCTAGAGCGCCGCTTTTTATTTCTTT.

The full-length 3′UTR of ROCK1 was amplified using cDNA from U87MG cells with following primers: TCTCTAGATTGTTCGTGCTTCCC and TCGAATTCATCAGTGCGGCTTTC. The 3′UTR was double-digested with XbaI/EcoRI and cloned downstream of firefly luciferase coding region sites of a modified pGL3-control plasmid.

### Transfection

The HEK293ET cells, U87MG cells and U251 cells were transfected with plasmids using Lipofectamine 2000 reagent (Invitrogen) at 24h after plating. Transfection complexes were prepared according to the manufacturer’s instructions. The transfection medium was replaced at 4h post-transfection.

### Bioinformatics Analysis

The target gene information of miR-124 was analyzed using miRanda (http://microrna.sanger.ac.uk/sequences/) and the Human microRNA targetscan (http://www.microrna.org/mammalian/index.html). The minimum free energy predicted for hybridization was determined by BibiServ analysis (http://bibiserv.techfak.uni-bielefeld.de/genefisher2/).

### Luciferase Reporter Assay

HEK293ET cells were co-transfected using lipofectamine 2000 reagent with 100ng of firefly luciferase construct and 300ng of control-pcDNA3.1 or pcDNA3.1-miR-124 expression vector. Ten ng of pRL-CMV (Promega) was co-transfected as a normalization control. Reporter assays were performed 48h post-transfection using the Dual-luciferase assay system (Promega), normalized for transfection efficiency by co-transfected Renilla luciferase. Cells were transfected in duplicated wells and such experiments were repeated three times on different days.

### Western Blot

Total protein samples were collected from U87MG cells 72h post-transfection with cell lysis buffer (Beyotime, Jiangsu, China). The protein concentration was determined by bicinchoninic acid protein assay kit (Beyotime). Heat-denatured protein samples (30 µg per lane) were resolved by 6%∼12% SDS-polyacrylamide gel electrophoresis and transferred to Hybond-nitrocellulose membranes (Amersham, Buckinghamshire, UK). The membrane was incubated for 2h in TBS containing 0.1% Tween 20 and 5% BSA to block nonspecific binding, followed by incubation for 12 h at 4°C with primary mouse monoclonal anti-ROCK1 antibody (Abcam, Cambridge, UK) (at 1∶1000 dilution). As a loading control, the GAPDH expression level was measured using mouse monoclonal anti-GAPDH antibody (Zsbio, Beijing, China), at 1∶1000 dilution. The membrane was then incubated with goat anti-mouse secondary antibody (Zsbio) using at 1∶5000 for 2h, then detected with SuperSignal West Pico Chemiluminescent Substrate (Thermo Scientific, Waltham, MA, USA) and visualized in an MF-ChemiBis 3.2 Bioimaging system (DNR, Jerusalem, Israel). Expression levels were quantified using ImageJ 1.44 software (National Institute of Health, Bethesda, MA, USA) and normalized to loading controls.

### Wound-healing Assay

Cells were plated at 80% confluence in DMEM supplemented with 10% FBS. At 24h after seeding, the monolayers were wounded by scoring with a sterile plastic 200 µl micropipette tip, washed, and then incubated in DMEM in the absence of serum. At vary hours, cells were photographed using a low-magnification fluorescence microscope (Olympus IX71, Miami, USA). The widths of the wound lines were measured by AlphaEase FC (Version 4.0, Alpha Innotech Corp.). The data are presented as percentages of the control.

### Invasion Assay

Cell invasion was assessed by the invasion of the cells through Matrigel-coated Transwell inserts. Briefly, Transwell inserts with 8 µm pore size were coated with 0.1ml of final concentration of 200 µg/ml Matrigel in cold serum-free medium. U87MG cells transfected were harvested 48 hours after treatment and trypsinized, then the serum-deprived cell suspension (200 µl; 0.5×10^5^ cells/ml) was added in triplicate to wells. After 24h incubation, cells that invaded the Matrigel and passed through the filter were stained with crystal violet and photographed using fluorescence microscope (Olympus IX71). The number of invaded cells was quantified by counting them in at least six random fields with total magnification of 100× per filter. In certain experiments, Y-27632 (10 µmol/L) was applied to the upper chamber.

### Fluorescence Microscopy

U87MG cells treated in 6-well plates were fixed in 4% formaldehyde for 10min and then washed three times for 10min each in PBS. The cells were then permeabilized with 0.1% Triton X-100 for 10min. After blocking with 5% BSA in PBS, the cells were incubated with TRITC-labeled phalloidin (Sigma-Aldrich) to stain the actin filaments. The nuclei were stained with DAPI. The coverslips were mounted and analyzed using fluorescence microscope (Olympus BX51).

### Scanning Electron Microscopy

U87MG cells were grown to sub confluence on 20mm square cover slip in a six-well plate. The cells were fixed with 2.5% glutaraldehyde in PBS (pH 7.4) for 2h at room temperature. Then, the cells were performed as described [69]. The specimens were cold sputter coated with gold and observed in a JSM-T300 scanning electron microscope (JEOL, Japan).

### Statistical Analysis

All tests were done using PRISM version 5.0 (GraphPad Software Inc., San Diego, CA, U.S.A.) statistical software. All experiments were carried out for three times independently. Data were mean±standard deviation except special indication. Spearman’s rank correlation test was used for association analysis between miR-124 and ROCK1 level data and pathological grading. Statistical significance between two groups was analyzed by Student’s t test. One-way ANOVA was used to compare multiple groups, with P<0.05 considered to be significant (*P<0.05; **P<0.01).

## Supporting Information

Figure S1 **Wound-healing assay of U251 glioma cells transfected with either control or the miR-124 expression vector, respectively.**
(TIF)

Figure S2 **qPCR assays of ROCK1 expression levels in glioma tissue samples.** The expression level of ROCK1 was downregulated significantly in low grade human glioma tissues (three grade I and five grade II) than that in high grade human glioma tissues (five grade III and three grade IV) determined using qRT–PCR.
(TIF)
