# Supplementary figures and images for: microRNA-124 Inhibits Migration and Invasion by Down-Regulating ROCK1 in Glioma
Source: PLoS One. 2013 Jul 23;8(7):e69478. doi: 10.1371/journal.pone.0069478 (PMC3720724; doi:10.1371/journal.pone.0069478)

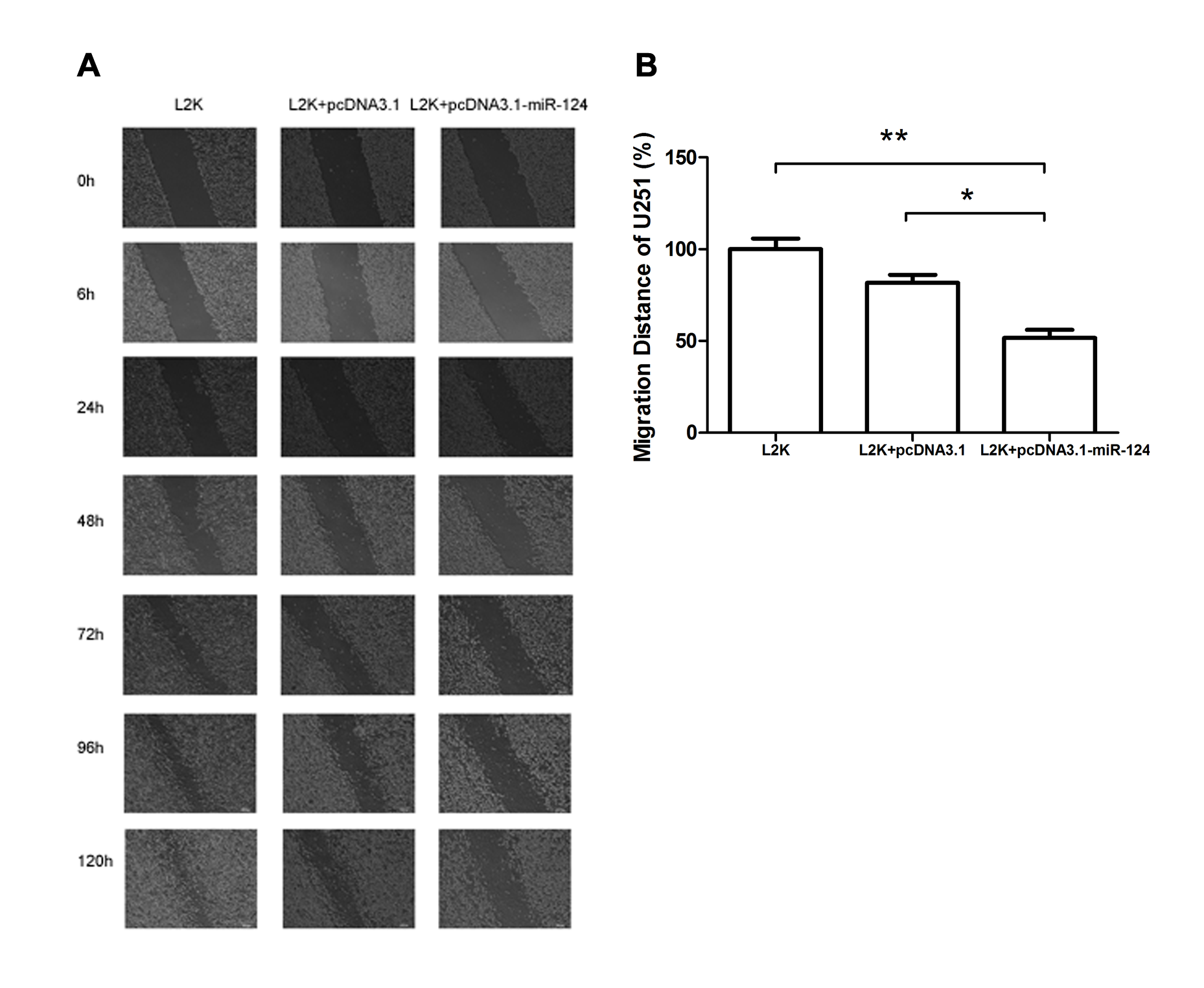

Supplement: Figure S1 — Wound-healing assay of U251 glioma cells transfected with either control or the miR-124 expression vector, respectively. (TIF) [file pone.0069478.s001.tif]

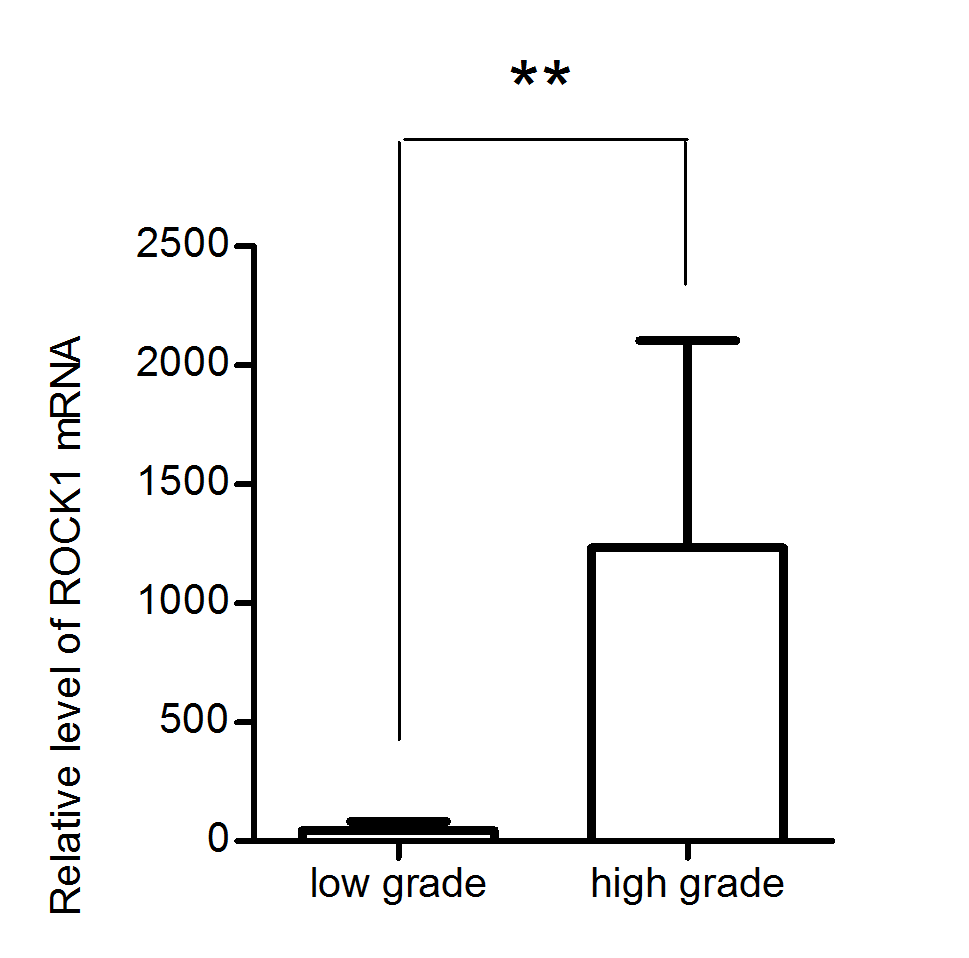

Supplement: Figure S2 — qPCR assays of ROCK1 expression levels in glioma tissue samples. The expression level of ROCK1 was downregulated significantly in low grade human glioma tissues (three grade I and five grade II) than that in high grade human glioma tissues (five grade III and three grade IV) determined using qRT–PCR. (TIF) [file pone.0069478.s002.tif]
